# Lifestyle Factors and Parkinson's Disease Risk in a Rural New England Case-Control Study

**DOI:** 10.1155/2021/5541760

**Published:** 2021-07-02

**Authors:** Angeline S. Andrew, Faith L. Anderson, Stephen L. Lee, Katharine M. Von Herrmann, Matthew C. Havrda

**Affiliations:** Geisel School of Medicine at Dartmouth, Rubin Building, Lebanon, NH, USA

## Abstract

**Introduction:**

Parkinson's disease (PD) is an age-related neurodegenerative disease likely caused by complex interactions between genetic and environmental risk factors. Exposure to pesticides, toxic metals, solvents, and history of traumatic brain injury have been implicated as environmental risk factors for PD, underscoring the importance of identifying risk factors associated with PD across different communities.

**Methods:**

We conducted a questionnaire-based case-control study in a rural area on the New Hampshire/Vermont border, enrolling PD patients and age- and sex-matched controls from the general population between 2017 and 2020. We assessed frequent participation in a variety of recreational and occupational activities and surveyed potential chemical exposures.

**Results:**

Suffering from “head trauma or a concussion” prior to diagnosis was associated with a fourfold increased risk of PD. Adjustment for head trauma negated any risk of participation in “strenuous athletic activities.” We observed a 2.7-fold increased risk of PD associated with activities involving lead (adjusted *p*=0.038).

**Conclusion:**

Implicating these factors in PD risk favors public health efforts in exposure mitigation while also motivating future work mechanisms and intervention opportunities.

## 1. Introduction

Parkinson's disease (PD) is a progressive neurodegenerative disease characterized by debilitating motor symptoms, including tremor, bradykinesia, and balance and mobility issues. Motor symptomatology results from a substantial loss of dopaminergic neurons in the substantia nigra of the brain, but factors involved in the initiation and progression of PD remain poorly characterized [1]. Twin studies indicated that genetic factors do not play a major role [2], only estimating to account for 5–10% of PD cases [3]. More recently, gene mapping in subjects with rare familial forms of PD [4] and large scale GWAS studies [5] have implicated numerous genetic variants associated with PD risk. Despite these discoveries, PD resulting from a causal genetic mutation is infrequent, suggesting the potential that lifestyle factors play a role in the development of the disease. Significant epidemiologic data suggest that environmental factors contribute substantially to PD etiology [6–8]. Identifying environmental factors that increase PD risk would allow exposure mitigation and disease prevention efforts while facilitating the experimental investigation of mechanisms and intervention opportunities.

Risk factors posited to increase the risk of PD include exposure to pesticides [9–17], toxic metals [16, 18–20], solvents [21, 22], and a history of traumatic brain injury (TBI) [23–26]. Many studies have found a positive association between rural living and the development of PD [27–29]. Numerous studies in agricultural cohorts have linked pesticide exposure to PD in farmers and pesticide applicators [15, 17, 27, 30–33]. A spatial analysis of US Medicare beneficiaries showed a concentration of PD in the Midwest, but also Northeast [34]. Agriculture and pesticide use are not prevalent in the Northeast, indicating the importance of analysis for additional lifestyle factors associated with PD in rural populations. To evaluate some of the risk factors in a rural location of the Northeast, we conducted a case-control study of PD based in the Dartmouth-Hitchcock Health System on the border of New Hampshire and Vermont. US. Questionnaires evaluated lifestyle factors, including a variety of specific jobs, hobby-related activities, and associated toxicant exposures.

## 2. Methods

Patients were enrolled and consented at the Dartmouth-Hitchcock Clinic (Lebanon, NH) within the Department of Neurology. We excluded patients with drug-induced parkinsonism. Patients were enrolled over a three-year period (April 2017–April 2020).

The population-control participants were identified as residents of New Hampshire or Vermont using the U.S. Postal Service Delivery Sequence file licensed to Marketing Systems Group (Horsham, PA). The sampling algorithm was designed based on the expected geographic and demographic distribution of the cases, focusing on Central and Northern New Hampshire/Vermont with oversampling of 50–75 year-olds and males. Questionnaires were mailed out to the sampled individuals, followed by a postcard reminder. Questionnaire response rates were 10% for the population controls. Participants who returned a completed questionnaire received a $20 reimbursement.

For the current analysis, we had eligible participant questionnaire data on *n* = 97 PD cases. We used the R-package “MatchIt” to perform propensity score matching with a 2 : 1 ratio to select a subset of questionnaires on *n* = 195 of the population controls as a comparison group with a similar distribution of age and sex to that of the cases [35]. This procedure selects two controls with the nearest age and same sex as each case.

We evaluated environmental risk factors using a questionnaire. We used our questionnaire to assess many activities, with the exploratory objective of identifying locally relevant potential risk factors that warrant future study in our rural New England population. Subjects reported if they participated in a variety of activities at least twice a month for a year or longer and answered questions about employment and hobbies. The following examples demonstrate the format of these questions: “Did you ever use paint strippers or thinners for at least 2 times each month for a year or longer?”; “Did you ever cast bullets or other lead objects for at least 2 times each month for a year or longer?”; “Did you make stained glass or art glass using lead joints for at least 2 times each month for a year or longer?”.

To assess physical activity, we asked, “Prior to the Diagnosis Date, did you ever participate regularly in strenuous athletic activities, such as running, swimming, or other competitive sports (soccer, football, etc.)? “We also assessed history of injuries, for example: “Have you ever suffered head trauma or a concussion that caused you to black out or lose consciousness?”; “Have you ever suffered a severe electrical burn or been electrocuted?”.

In addition to exposure and physical activity, participants reported on the family medical history of “diagnosis of neurological disorder” (Parkinson's, Dementia or Alzheimer's, ALS, Multiple Sclerosis, or other) within each of their family members (Mother, Father, Brother or Sister, Child, Spouse, Maternal Grandparent, Paternal Grandparent, Maternal Aunt/Uncle, Paternal Aunt/Uncle).

Participants provided written consent to join the study. This study is reviewed, approved, and is overseen by the Committee for Protection of Human Subjects, the Institutional Review Board of Dartmouth College (CPHS #: STUDY00030209).

Our statistical analysis began by testing for univariate associations between the response variable, case-control status of the participants, and categorical predictor variables by utilizing the chi-square test of independence and Fisher's exact test (Table 1). We used the risk of PD associated with a specific exposure or lifestyle factor identified in the univariate analyses in the subsequent multivariable analysis described below. Multivariable modeling used case-control status as the outcome in an unconditional logistic regression analysis unadjusted, then with adjustment for the potential confounders of age, sex, family history, and smoking status, and also a model adding an adjustment for athletics and head trauma (Table 2). Statistically significant findings are defined as*p* < 0.05. The index year was defined as the year of diagnosis for PD patients or an equivalent year for controls. These analyses were all performed using R:A Language and Environment for Statistical Computing, version 3.6.3 (R Foundation for Statistical Computing, Vienna, Austria).

## 3. Results

Table 1 shows the demographic characteristics of PD patients and controls. Due to the matched case-control study design, there were no significant differences in the age or sex distributions. A higher proportion of cases had a family history of PD in a first or second-degree relative (22.7%), compared to 6.7% of the controls (*p* < 0.001). Smoking histories were similar for cases and controls, with 44.2% and 48.2% reporting ever smoking more than 100 lifetime cigarettes, respectively (*p*=0.613).

The questionnaire asked about athletic activities and injuries. A higher proportion of PD patients reported participation in “strenuous athletic activities, such as running, swimming, or other competitive sports (soccer, football, etc.)” (57.9% of cases vs. 43.2% of controls, *p*=0.020); however, adjusting for head trauma as a confounding factor mitigates this association (OR 1.69 95% CI 0.93–3.13) (Table 2). Reports of suffering “head trauma or a concussion that caused you to black out or lose consciousness” prior to the diagnosis date were much more frequent among PD patients (42.7%) compared to controls (18.4%). The risk remained significantly increased in a model adjusted for age, sex, smoking, family history, and athletic activity (OR 4.17 95% CI 2.23–7.98) (Table 2). In contrast, having “ever suffered a severe electrical burn or been electrocuted” was not associated with PD (*p*=1.00) (Table 2).

Our questionnaire also collected data on numerous job- or hobby-related activities that occurred at least twice a month for a year or longer.  Table 3 addresses the hypothesis that activities involving lead exposure would increase PD risk. These activities included “making stained glass or art glass using lead joints,” “casting bullets or other lead objects,” and “making or using lead fish weights/sinkers.” Considered together, we observed a 2.67-fold increased risk of PD associated with these activities, adjusted for age, sex, smoking, head trauma, and family history (*p*=0.038).

Supplemental Table 1 shows the results of our exploratory analysis of other activities, with several that showed ≥1.5-fold ratio of exposed PD cases vs. controls but did not meet our statistical significance threshold. For example, the use of “pastels or pigments” and “welding, soldering, brazing or tinning” were activities modestly associated with PD. Self-reported use of herbicides and insecticides were also modestly more common among cases compared to controls. Overall, only 13% of our population reported being exposed to either type of pesticide (16% of cases, 11% of controls, *p*=0.212).

Numerous exposures did not appear to differ between the case and control groups of our population, however. Overall, approximately 9–10% of respondents reported having “participated in farming/agriculture for at least two times each month for a year or longer,” but this was not associated with PD. Similarly, 4–6% were involved in “animal husbandry,” which was unrelated to PD status. Nearly half of those surveyed frequently “participated in gardening or lawn care” with little difference between cases and controls. We saw no evidence of a link between PD and repairing or restoring cars, trapping or shooting, or burning trash. Carpentry, home remodeling, and electrical work were not associated with PD.

## 4. Discussion

It is widely accepted that PD has an environmental etiology, but the risk factors remain unclear. Identifying these factors is important for disease prevention and also for elucidating mechanisms and potential drug targets. In our Northern New England case-control study, we assessed a variety of lifestyle and behavioral factors in relation to PD risk using a detailed questionnaire.

After adjusting for a head injury, physical activity did not increase PD risk, suggesting that sports related concussive events are likely acting as a confounder in this relationship. In fact, moderate to vigorous activity at ages 35–39 was associated with a lower risk of developing PD in the NIH-AARP Diet and Health Study Cohort [36]. TBI is a suspected PD risk factor. However, the link is not definitive. The increased risk that we observed is consistent with the pooled OR 1.57 (95% CI 1.35–1.83) found by a 2013 systematic review of 22 studies [23]. The magnitude of the risk we observed (four-fold) may be explained in part by some amount of reverse causality. Prior to diagnosis, some patients may have early experienced symptoms of postural instability that led to a higher propensity to falls.

Several prospective studies in the literature have assessed the link between TBI and the development of PD. A U.S. prospective cohort of 7130 individuals studied TBI with loss of consciousness and subsequent neuropathic outcomes. They found a strong association between TBI and subsequent incident PD (HR 3.56 95% CI 1.52–8.28), with 117 cases identified [37]. Similarly, a California study followed patients injured ≥ age 55, without PD or dementia prospectively over time. The patients in that cohort with TBI had a 1.7-fold increased risk of subsequently developing PD, which was dose-responsive to both the severity and frequency of injury [24]. A study of military veterans found a similar risk 1.71 (95% CI 1.53–1.92), with higher risks associated with more severe injuries [25]. In contrast, a Danish case-control study of 1785 patients used medical records to assess head injury before onset of “first cardinal symptom” and found no association [26]. In terms of future directions, the mechanisms linking head injuries to PD risk remain understudied. In this context, it is noteworthy that inflammation continues to be implicated in PD [38, 39], suggesting a possible link between brain injury and eventual neurodegeneration.

Animal studies also support the link between PD and head injury. Expression of the dopamine transporter protein was disrupted in the midbrain of mice during the two weeks following TBI [40]. Three mild head injuries led to changes in connectivity in the midbrain dopaminergic and striatum that was still visible in rodents by multimodal MRI after two months [41]. Similarly, after one month, a moderate midline fluid percussion brain injury caused a loss of 44% of the neurons in the substantia nigra of rats [42]. Delic et al. recently reviewed biological mechanisms that could explain this link, including the accumulation of proteins including alpha-synuclein following TBI, inflammation, and metabolic dysregulation [43]. TBI also induces upregulation of Leucine-Rich Repeat Kinase 2 (LRRK2), and blocking LRRK2 is neuroprotective in both TBI and PD models [43].

We also observed a 2.7-fold increased risk of PD associated with participating in activities involving lead. These findings are consistent with a case report that found Parkinsonism in seven out of nine charging station workers with 30 years of lead-sulfate battery exposure that began in 1947 [44, 45]. Findings from several biomarker-based assessments of the link between lead exposure and PD also support our results. A case-control study in Michigan also found a significantly higher risk of PD among participants in the top quartile for lead exposure assessed using occupational history and bone lead levels (adjusted odds ratio 2.27 (95% CI 1.13–4.55), *p*=0.021) [46]. A study in Massachusetts also found an elevated adjusted odds ratio of 3.21 (95% CI 1.17–8.83) for the highest vs. lowest quartile of tibia bone lead in PD patients vs. controls [18].

Animal studies demonstrate the transport of lead ions across the blood-brain barrier [47]. Rats exposed to lead had tau hyperphosphorylation and accumulation of alpha-synuclein, which induced ER stress and suppressed the mTOR signaling pathway [48]. Early-life lead exposure also increased alpha-synuclein levels in aged tau-knockout mice [49]. Although efforts to mitigate lead exposure have been successful, our findings and the continuing potential for lead exposures resulting from deteriorating urban infrastructure highlight the importance of PD risk assessment in aging populations potentially exposed to lead. Monitoring of populations with documented lead exposures, such as those in Flint, Michigan 2014–2015 and Washington, DC, 2000–2004, could enable early disease detection [50, 51].

In our study, family history of PD in a first- or second-degree relative was associated with fourfold risk, which is higher than reported in the UK Biobank cohort (adjusted OR 2.19 95% CI 1.81–2.65) [52]. Some of the subjects we identified likely carried genetic alterations associated with familial PD, but the likelihood that this is a highly prevalent feature of our population is low given the infrequency of such genetic alterations in the general population. Our multivariable models included adjustment for a family history of PD since family members may also share exposure profiles based on lifestyle commonalities.

We observed nonsignificant trends with activities involving “pastels or pigments,” as well as PD risk factors previously reported in larger studies, including “welding, soldering, brazing or tinning,” and use of insecticides. The prevalence of parkinsonism among shipyard and fabrication welders was 15.6%, compared to 0% in nonwelder trade workers [53]. In addition, soldering and welding are both potential sources of lead exposure. However, we did not have information on the specific materials used in these activities [54].

Our study in a rural New Hampshire/Vermont cohort found no association between generally participating in animal husbandry, farming/agriculture, or gardening/lawn care activities. Pesticide exposure was more common among cases than controls; however, the overall prevalence of use in this Northern New England population is only 13%, limiting our statistical power to detect the difference. The use of certain pesticides has clearly been prospectively associated with the development of PD in the Agricultural Health Study cohort of 52,000 pesticide applicators in North Carolina and Iowa, particularly the mitochondrial inhibitor rotenone (OR 2.5 95% CI 1.3–4.7), paraquat (OR 2.0 95% CI 1.2–3.6), which is associated with oxidative stress [55], and the organophosphate insecticide Terbufos (HR 1.31 95% CI 1.02–1.68) [31].

Limitations of our study include small numbers of cases for the analysis of subgroups of certain exposure factors and lower questionnaire response rates. Each of the associated factors reported was unrelated to the disease duration or to dementia (*p* > 0.1). The demographics of those who answered the questionnaire were similar to the nonrespondents. Nevertheless, patients may be more likely to recall life events (e.g., head injury) due to thinking about what may have caused their illness.

Overall, our results from Northern New England support previous reports of head injury and lead exposure as PD risk factors. From a public health perspective, our findings and the literature encourage the use of lead substitutes and helmets, which are general health recommendations that may also reduce the risk of PD.

## Figures and Tables

**Table 1:** Population characteristics and univariate associations.

		Controls	PD	Univariate^*∗*^
		*n* = 195 (%)	*n* = 97 (%)	*p*-value
Sex	F	78 (40.0)	34 (35.1)	0.489
	M	117 (60.0)	63 (64.9)	

Age	Mean ± SD	67.6 ± 7.29	69.36 ± 8.10	0.072

Age category	<65	70 (35.9)	31 (32.0)	0.411
	65–75	100 (51.3)	48 (49.5)	
	75+	25 (12.8)	18 (18.6)	

Family history of PD	No	182 (93.3)	75 (77.3)	<0.001
	Yes	13 (6.7)	22 (22.7)	

Disease duration	Mean ± SD		6.57 ± 5.26	

Dementia or Alzheimer's	No	195 (0.0)	94 (98.9)	1
	Yes	0 (0.0)	1 (1.1)	

Smoking	Never	99 (51.8)	53 (55.8)	0.613
	Ever	92 (48.2)	42 (44.2)	

Participate regularly in strenuous athletic activities, such as running, swimming, or other competitive sports (soccer, football, etc.)	No	108 (56.8)	40 (42.1)	0.026
	Yes	82 (43.2)	55 (57.9)	
				

Ever suffered head trauma or a concussion that caused you to black out or lose consciousness?	No	155 (81.6)	55 (57.3)	<0.001
	Yes	35 (18.4)	41 (42.7)	
				

Have you ever suffered a severe electrical burn or been electrocuted?	No	182 (96.3)	90 (95.7)	1
	Yes	7 (3.7)	4 (4.3)	

^*∗*^Chi-square or *t*-test.

**Table 2:** PD risk related to physical activities and injuries.

		Controls	PD	Univariate logistic model	Adjusted^*∗*^ model	Adjusted^*∗*^ model, plus athletics and head trauma
		*n* = 195	*n* = 97			
Participate regularly in strenuous athletic activities, such as running, swimming, or other competitive sports (soccer, football, etc.)	No	108 (56.8)	40 (42.1)	OR 1.81 (95% CI 1.10–2.99)	OR 2.12 (95% CI 1.19–3.81)	OR 1.69 (95% CI 0.93–3.13)
	Yes	82 (43.2)	55 (57.9)	*p*=0.020	*p*=0.011	*p*=0.087

Ever suffered head trauma or a concussion that caused you to black out or lose consciousness?	No	155 (81.6)	55 (57.3)	OR 3.30 (95% CI 1.92–5.73)	OR 4.05 (95%CI 2.22–7.53)	OR 4.17 (95% CI 2.23–7.98)
	Yes	35 (18.4)	41 (42.7)	*p* < 0.001	*p*=7.04*E* − 6	*p*=0.000011

Have you ever suffered a severe electrical burn or been electrocuted?	No	182 (96.3)	90 (95.7)	OR 1.15 (95% CI 0.30–3.93)	OR 1.02 (95% CI 0.24–3.81)	
	Yes	7 (3.7)	4 (4.3)	*p*=1.0	*p*=0.971	

^*∗*^Adjusted for age, sex, smoking, family history.

**Table 3:** Use of lead in jobs or hobbies and PD risk.

At least 2 times each month for a year or longer:		Controls	PD	Logistic regression model
		*n* = 195	*n* = 97	Univariate
Make a stained glass or art glass using lead joints	No	192 (98.5)	93 (95.9)	OR 2.75 (95% CI 0.60–14.21)
	Yes	3 (1.5)	4 (4.1)	*p*=0.191
Cast bullets or other lead objects	No	191 (97.9)	97 (100.0)	NA
	Yes	4 (2.1)	0 (0.0)	*p*=0.984
Make or use lead fish weights/sinkers	No	188 (96.4)	87 (89.7)	OR 3.09 (95% CI 1.15–8.76)
	Yes	7 (3.6)	10 (10.3)	*p*=0.027
Any of above hobbies	No	182 (93.3)	85 (87.6)	OR 3.09 (95% CI 1.15–8.76)
	Yes	13 (6.7)	12 (12.4)	*p*=0.106
				Multivariable^*∗*^
				^*∗*^OR 2.67 (95% CI 1.05–6.80)
				^*∗*^ *p*=0.038

^*∗*^Adjusted for age, sex, smoking, head trauma, family history.

## Data Availability

The questionnaire and health data used to support the findings of this study are restricted by the Dartmouth Committee for Protection of Human Subjects in order to protect patient privacy. Data are available from Matthew Havrda (matthew.havrda@dartmouth.edu) for researchers who meet the criteria for access to confidential data.

## Supplementary Materials

Supplementary Materials Supplemental Table 1 contains a list of activities and exposures collected using our questionnaire. We compared responses among PD patients vs. controls using a chi-square test to identify potential associations for further study.
