# Supplementary material for: Lifestyle Factors and Parkinson's Disease Risk in a Rural New England Case-Control Study
Source: Parkinsons Dis. 2021 Jul 2;2021:5541760. doi: 10.1155/2021/5541760 (PMC8270694; doi:10.1155/2021/5541760)
Supplement: Supplementary Materials — Supplemental Table 1 contains a list of activities and exposures collected using our questionnaire. We compared responses among PD patients vs. controls using a chi-square test to identify potential associations for further study. [file 5541760.f1.docx]

| **Supplemental Table 1.** Exposure questions and PD risk. |  |  |  |  |
| --- | --- | --- | --- | --- |
|  |  | **Controls** | **PD** | **univariate** |
|  |  | n=195 | n=97 | **p-value** |
| Did your job(s) or hobbies involve exposure to potentially harmful chemicals? | No | 109 (59.9) | 45 (47.4) | 0.062 |
|  | Yes | 73 (40.1) | 50 (52.6) |  |
| Lead | No | 180 (92.3) | 87 (89.7) | 0.596 |
|  | Yes | 15 (7.7) | 10 (10.3) |  |
| Mercury | No | 188 (96.4) | 90 (92.8) | 0.282 |
|  | Yes | 7 (3.6) | 7 (7.2) |  |
| Solvents (paint thinners, degreasers, methanol) | No | 154 (79.0) | 76 (78.4) | 1.000 |
|  | Yes | 41 (21.0) | 21 (21.6) |  |
| Cooling, Cutting or Lubricating Oils | No | 178 (91.3) | 87 (89.7) | 0.820 |
|  | Yes | 17 (8.7) | 10 (10.3) |  |
| Herbicides | No | 176 (90.3) | 83 (85.6) | 0.319 |
|  | Yes | 19 (9.7) | 14 (14.4) # |  |
| Insecticides | No | 180 (92.3) | 84 (86.6) | 0.177 |
|  | Yes | 15 (7.7) | 13 (13.4) # |  |
| Did you ever repair or restore cars (other than fixing a flat tire or changing the oil) for at least 2 times each month for a year or longer? | No | 175 (89.7) | 87 (89.7) | 1.000 |
|  | Yes | 20 (10.3) | 10 (10.3) |  |
| Have you ever participated in Welding, Soldering, Brazing or Tinning for at least 2 times each month for a year or longer? | No | 178 (91.3) | 82 (84.5) | 0.124 |
|  | Yes | 17 (8.7) | 15 (15.5) # |  |
| Have you ever participated in Carpentry at least 2 times each month for a year or longer? | No | 162 (83.1) | 79 (81.4) | 0.855 |
|  | Yes | 33 (16.9) | 18 (18.6) |  |
| Have you ever participated in Home remodeling at least 2 times each month for a year or longer? | No | 150 (76.9) | 73 (75.3) | 0.866 |
|  | Yes | 45 (23.1) | 24 (24.7) |  |
| Have you ever participated in Electrical work at least 2 times each month for a year or longer? | No | 182 (93.3) | 89 (91.8) | 0.801 |
|  | Yes | 13 (6.7) | 8 (8.2) |  |
| Did you ever use paint strippers or thinners for at least 2 times each month for a year or longer? | No | 173 (88.7) | 83 (85.6) | 0.560 |
|  | Yes | 22 (11.3) | 14 (14.4) |  |
| Have you ever participated in Metal work for at least 2 times each month for a year or longer? | No | 186 (95.4) | 91 (93.8) | 0.771 |
|  | Yes | 9 (4.6) | 6 (6.2) |  |
| Have you ever participated in Amalgamation work for at least 2 times each month for a year or longer? | No | 192 (98.5) | 97 (100.0) | 0.541 |
|  | Yes | 3 (1.5) | 0 (0.0) |  |
| Have you ever participated in Animal husbandry for at least 2 times each month for a year or longer? | No | 184 (94.4) | 93 (95.9) | 0.786 |
|  | Yes | 11 (5.6) | 4 (4.1) |  |
| Have you ever participated in Farming/Agriculture for at least 2 times each month for a year or longer? | No | 177 (90.8) | 87 (89.7) | 0.933 |
|  | Yes | 18 (9.2) | 10 (10.3) |  |
| Did you trap, hunt, or shoot (animals, target, or skeet) for at least 2 times each month for a year or longer? | No | 172 (88.2) | 86 (88.7) | 1.000 |
|  | Yes | 23 (11.8) | 11 (11.3) |  |
| Have you ever participated in Gardening or lawn care for at least 2 times each month for a year or longer? | No | 106 (54.4) | 50 (51.5) | 0.742 |
|  | Yes | 89 (45.6) | 47 (48.5) |  |
| Did you burn trash for at least 2 times each month for a year or longer? | No | 184 (94.4) | 92 (94.8) | 1.000 |
|  | Yes | 11 (5.6) | 5 (5.2) |  |
| Have you ever participated in Oil painting (fine art) for at least 2 times each month for a year or longer? | No | 188 (96.4) | 91 (93.8) | 0.477 |
|  | Yes | 7 (3.6) | 6 (6.2) |  |
| Did you use pastels or pigments at least 2 times each month for a year or longer? | No | 190 (97.4) | 89 (91.8) | 0.055 |
|  | Yes | 5 (2.6) | 8 (8.2) # |  |
| Did you make silver jewelry at least 2 times each month for a year or longer? | No | 194 (99.5) | 96 (99.0) | 1.000 |
|  | Yes | 1 (0.5) | 1 (1.0) |  |
| Did you make pottery or ceramics for at least 2 times each month for a year or longer? | No | 193 (99.0) | 94 (96.9) | 0.422 |
|  | Yes | 2 (1.0) | 3 (3.1) |  |
| * Statistically significant (*P*<0.05) |  |  |  |  |
| # Exposed PD vs. control ≥1.5-fold |  |  |  |  |
